# Supplementary material for: A Combinatorial Code for CPEB-Mediated c-myc Repression
Source: Cells. 2023 Oct 6;12(19):2410. doi: 10.3390/cells12192410 (PMC10572585; doi:10.3390/cells12192410)
Supplement: Supplementary file 1 [file cells-12-02410-s001.zip › cells-2420937-supplementary.pdf]

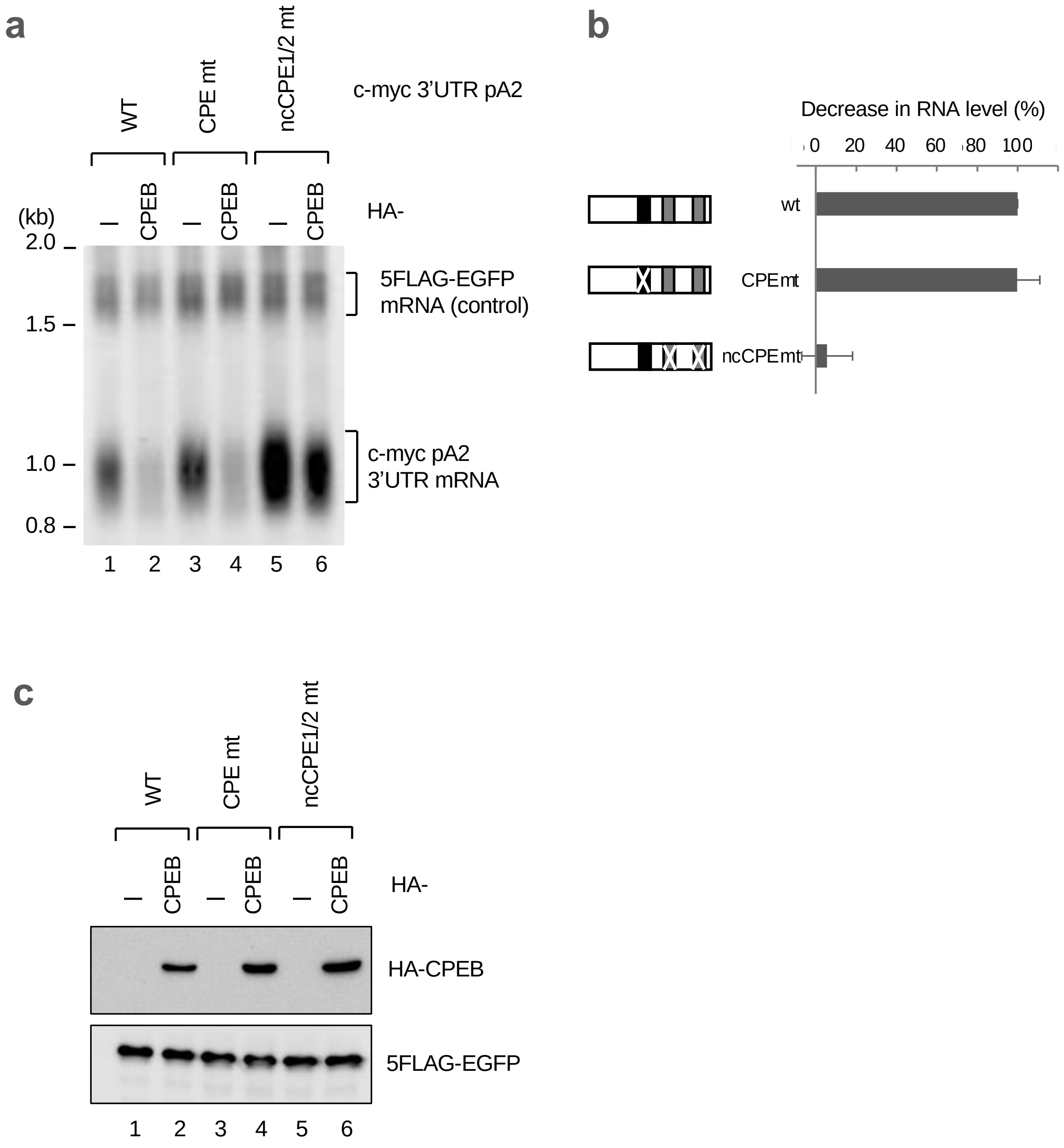

Figure S1. Conservation of CPEB-mediated mRNA decay in IMR-90 cells. (a) IMR-90 cells were transfected with pFlag-CMV5/TO-BGG c-myc 3'UTR pA2 or its point mutation reporter plasmid, pCMV-5×Flag-EGFP reference plasmid, and either pHA-CMV5-CPEB or pHA-CMV5. Total RNA was prepared from the cells and subjected to Northern blotting. (b) Schematic diagram of c-myc 3'UTR pA2 mutants (left). The cCPEs and ncCPEs are depicted as black and gray boxes, respectively. The cross bars on the boxes represents the mutated motifs. Relative BGG mRNA levels in CPEB-overexpressed cells, as in (a), were estimated and normal-ized by 5×FLAG-EGFP mRNA (right). The data are presented as the mean ± SD (n = 3). (c) Western blotting showing the expression of HA-CPEB, as in (a). 5FLAG-EGFP was used as a transfection control.

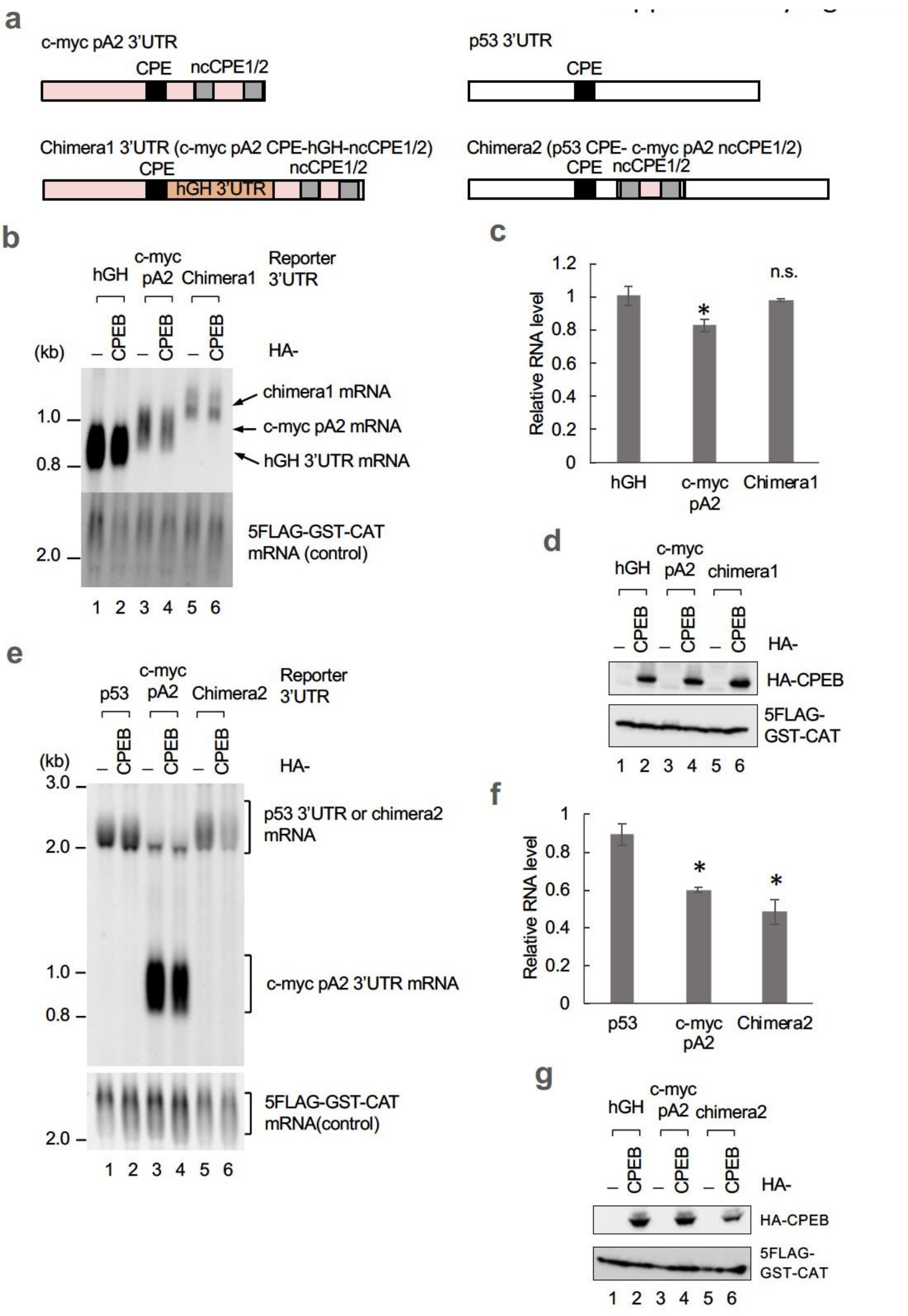
a
c-myc pA2 3'UTR
CPE
ncCPE1/2
p53 3'UTR
CPE
Chimera1 3'UTR (c-myc pA2 CPE-hGH-ncCPE1/2)
CPE
hGH 3'UTR
ncCPE1/2
Chimera2 (p53 CPE- c-myc pA2 ncCPE1/2)
CPE
ncCPE1/2
b
hGH
c-myc pA2
Chimera1
Reporter 3'UTR
(kb)
– CPEB – CPEB – CPEB
HA-
1.0
0.8
2.0
chimera1 mRNA
c-myc pA2 mRNA
hGH 3'UTR mRNA
5FLAG-GST-CAT mRNA (control)
1 2 3 4 5 6
c
Relative RNA level
1.2
1
0.8
0.6
0.4
0.2
0
*
n.s.
hGH
c-myc pA2
Chimera1
d
hGH
c-myc pA2
chimera1
– CPEB – CPEB – CPEB
HA-
HA-CPEB
5FLAG-GST-CAT
1 2 3 4 5 6
e
p53
c-myc pA2
Chimera2
Reporter 3'UTR
(kb)
– CPEB – CPEB – CPEB
HA-
3.0
2.0
1.0
0.8
2.0
p53 3'UTR or chimera2 mRNA
c-myc pA2 3'UTR mRNA
5FLAG-GST-CAT mRNA(control)
1 2 3 4 5 6
f
Relative RNA level
1
0.8
0.6
0.4
0.2
0
*
*
p53
c-myc pA2
Chimera2
g
hGH
c-myc pA2
chimera2
– CPEB – CPEB – CPEB
HA-
HA-CPEB
5FLAG-GST-CAT
1 2 3 4 5 6

Figure S2. A tandemly arranged cCPE and ncCPE constitute a combinatorial code for CPEB-mediated mRNA decay. (**a**) Schematic diagram of c-myc pA2 3'UTR, Chimera1 3'UTR (c-myc pA2 CPE-hGH-ncCPE1/2), p53 3'UTR and Chimera2 (p53 CPE-c-myc pA2 ncCPE1/2). The consensus CPEs and non-consensus CPEs are depicted as black and gray boxes, respectively. The pink bars represent the c-myc pA2 3'UTR derived sequences, the red bar represents hGH 3'UTR derived sequence and the white bars represent the p53 3'UTR derived sequences. (**b**)HeLa cells infected with HA-CPEB-expressing or empty lentiviruses were transfected with a reference plasmid pCMV-5×Flag-GST-CAT and either pFlag-CMV5/TO-BGG c-myc pA2 3'UTR, pFlag-CMV5/TO-BGG Chimera1 3'UTR (c-myc pA2 CPE-hGH-ncCPE1/2) or pFlag-CMV5/TO-BGG hGH 3'UTR. Total RNA was prepared from the cells and subjected to Northern blotting. (**c**) Relative BGG mRNA levels in CPEB-overexpressed cells as in (**b**) were estimated and normalized by GST-CAT mRNA. Data are the mean ± s.d. (n=3). (**d**) Western blotting showing the expression of HA-CPEB as in (**b**). 5FLAG-GST-CAT was served as a transfection control. (**e**)HeLa cells were transfected with pFlag-CMV5/TO-BGG c-myc pA2 3'UTR, pFlag-CMV5/TO-BGG Chimera2 3'UTR (p53 CPE-c-myc pA2 ncCPE1/2) or pFlag-CMV5/TO-BGG p53 3'UTR, pCMV-5×Flag-GST-CAT reference plasmid, and either pHA-CMV5-CPEB or pHA-CMV5. Total RNA was prepared from the cells and subjected to Northern blotting. (**f**) Relative BGG mRNA levels in CPEB-overexpressed cells as in (**e**) were estimated and normalized by GST-CAT mRNA. Data are the mean ± s.d. (n=3). (**g**) Western blotting showing the expression of HA-CPEB as in (**e**). 5FLAG-GST-CAT was served as a transfection control.

Table S1. List of templates and primers to construct reporter plasmids.

Resulting plasmid	primer1	primer2	template
pFlag-CMV5/TO-BGG c-myc 3'UTR pA1	AAT AAA ATA ACT GGC AAA TAT ATC ATT GAG CCA AAT	AAA GTT ATT TAC ATT TAA TGG CAA TAT TTA CAG AGA	pFlag-CMV5/TO-BGG c-myc 3'UTR (Ogami et al., Oncogene 2014)
pFlag-CMV5/TO-BGG c-myc 3'UTR pA2	ACG TTT ATA GCA GTT ACA CAG AAT TTC AAT CCT AGT	CTT AGT GAT ACT TGT GGG CCA GGG	pFlag-CMV5/TO-BGG c-myc 3'UTR (Ogami et al., Oncogene 2014)
pFlag-CMV5/TO-BGG c-myc 3'UTR pA2 CPE mt	CTA ATT TTT TGG ATT TAA GTA CAT TTT GCT	ACT TAA ATC CAA AAA ATT AGG GTT TAT AGT	pFlag-CMV5/TO-BGG c-myc 3'UTR pA2
pFlag-CMV5/TO-BGG c-myc 3'UTR pA2 ncCPE1 mt	TTT CTA TTG TTT TTA GAA AAA ATA AAA TAA	AAA ATC AAC TTT CCA AAG CAA AAT GTA CTT	pFlag-CMV5/TO-BGG c-myc 3'UTR pA2
pFlag-CMV5/TO-BGG c-myc 3'UTR pA2 ncCPE2 mt	TTT CTA TTG TTT GGA GAA AAA ATA AAA TAA	AAA ATC AAC TTT AAA AAG CAA AAT GTA CTT	pFlag-CMV5/TO-BGG c-myc 3'UTR pA2
pFlag-CMV5/TO-BGG c-myc 3'UTR pA1 CPE mt	AAG AAC TTT TGG ATG CTT ACC ATC TTT TTT	GTA AGC ATC CAA AAG TTC TTT TAT GCC CAA	pFlag-CMV5/TO-BGG c-myc 3'UTR pA1
pFlag-CMV5/TO-BGG c-myc 3'UTR pA1 ncCPE1/2 mt	ACA ATG TTT CTC TGT AAA TAT TGC CAT TAA	GTA AAT CTT CCA ATT TTT TCC AAA CAA TTC	pFlag-CMV5/TO-BGG c-myc 3'UTR pA1
pFlag-CMV5/TO-BGG c-myc 3'UTR pA2 5'D1	CCC TAA TTT TTT TTA TTT AAG TAC ATT TTG CTT TTT	CTT AGT GAT ACT TGT GGG CCA GGG	pFlag-CMV5/TO-BGG c-myc 3'UTR pA2
pFlag-CMV5/TO-BGG c-myc 3'UTR pA2 5'D2	CAT TTT GCT TTT TAA AGT TGA TTT TTT TCT ATT GTT	CTT AGT GAT ACT TGT GGG CCA GGG	pFlag-CMV5/TO-BGG c-myc 3'UTR pA2
pFlag-CMV5/TO-BGG c-myc 3'UTR pA2 5'D3	GTT GAT TTT TTT CTA TTG TTT TTA GAA AAA ATA AAA	CTT AGT GAT ACT TGT GGG CCA GGG	pFlag-CMV5/TO-BGG c-myc 3'UTR pA2
pFlag-CMV5/TO-BGG c-myc 3'UTR pA2 3'D1	AAT AAA ATA ACT GGC AAA TAT ATC ATT GAG CCA AAT	CTT TAA AAA GCA AAA TGT ACT TAA ATA AAA	pFlag-CMV5/TO-BGG c-myc 3'UTR pA2
pFlag-CMV5/TO-BGG c-myc 3'UTR pA2 3'D2	AAT AAA ATA ACT GGC AAA TAT ATC ATT GAG CCA AAT	CTT AAA TAA AAA AAA TTA GGG TTT ATA GTA	pFlag-CMV5/TO-BGG c-myc 3'UTR pA2
pFlag-CMV5/TO-BGG hGH 3'UTR	CGG GTA AGC TTC CTG TGA CCC CTC CCC AGT	TTT AAC TGC AGA AGG ACA GGG AAG GGA GCA	pHA-CMV5 (Ogami et al., Oncogene 2014)
pFlag-CMV5/TO-BGG c-jun 3'UTR	CAG TTG CAA AGC TTT TCT AGA GAG ACC GTC	TCT GGG CTT AGT CTA CCT CTG CAG GTA ATT	genomic DNA
pFlag-CMV5/TO-BGG p53 3'UTR	GAC TCA AGC TTA CAT TCT CCA CTT CTT	CAA TTC ATT CAG GTA CCT GGC ATG AGG	genomic DNA
pCDH-EF1-HA-hCPEB1	GAC CGG CGC CTA CTC TAG AGC TAG CGC CAC CAT GTA CCC ATA CGA CGT	CCG ATT TAA ATT CGA ATT CGC TAG CTG GAA TCT CGG TTC TTC TGG	pHA-CPEB
